# Heat and charge transport in H_2_O at ice-giant conditions from ab initio molecular dynamics simulations

**DOI:** 10.1038/s41467-020-17275-5

**Published:** 2020-07-17

**Authors:** Federico Grasselli, Lars Stixrude, Stefano Baroni

**Affiliations:** 1 SISSA—Scuola Internazionale Superiore di Studi Avanzati, Trieste, Italy; 2 Department of Earth, Planetary, and Space Sciences, University of California Los Angeles, Los Angeles, USA; 3 CNR—Istituto Officina dei Materiali, SISSA, Trieste, 34136 Italy; 4 Present Address: COSMO – Laboratory of Computational Science and Modelling, IMX, École Polytechnique Fédérale de Lausanne (EPFL), Lausanne, 1015 Switzerland

**Keywords:** Planetary science, Condensed-matter physics

## Abstract

The impact of the inner structure and thermal history of planets on their observable features, such as luminosity or magnetic field, crucially depends on the poorly known heat and charge transport properties of their internal layers. The thermal and electric conductivities of different phases of water (liquid, solid, and super-ionic) occurring in the interior of ice giant planets, such as Uranus or Neptune, are evaluated from equilibrium ab initio molecular dynamics, leveraging recent progresses in the theory and data analysis of transport in extended systems. The implications of our findings on the evolution models of the ice giants are briefly discussed.

## Abstract (web summary)

The authors here perform ab initio calculations to investigate in the heat transport properties of water at extreme pressure and temperature conditions, typically found in the interior of ice giants such as Uranus and Neptune.

## Introduction

Hydrogen and oxygen are two of the three most abundant elements in the universe, helium being the second. As a consequence, H_2_O is thought to be a major constituent of celestial bodies formed far enough from their host star for it to condense^1^. Many moons of the outer solar system, such as Ganymede, Europa, and Enceledus, have rigid icy shells and interior water oceans, which are key for understanding the observed surface mass flux^2^ and the generation of magnetic fields^3^. The ice giants, Uranus and Neptune, are thought to be composed primarily of H_2_O^4^: throughout most of their interior, the large pressure and temperature (e.g., 240 GPa and 5000 K at half the radius of Uranus) favor a super-ionic (SI) phase, where oxygen ions are arranged in a crystalline lattice and protons diffuse freely like in a fluid^5,6^. Partially dissociated, liquid (PDL) water may instead be confined to the outermost third of the interior, where the magnetic field is generated^7^. Outside the solar system, the observed characteristics of many exoplanets are also consistent with with water-rich interiors^8^.

Our knowledge of the interior of planets other than Earth mostly relies on the observation of their magnetic fields and surface properties, which are affected by the inner structure through the transport of energy, mass, and charge across intermediate layers. In the case of Uranus, for instance, it has long been recognized that its remarkably small luminosity^9^ can be explained by nonadiabatic models of the interior^4,10^, featuring thermal boundary layers whose transport properties are poorly known. Likewise, any model aiming to explain the anomalous multipolar and non-axisymmetry magnetic fields of Uranus and Neptune requires the knowledge of the electric conductivity of the various phases of water occurring in their interior^11^. More generally, a detailed knowledge of the transport properties of different phases of H_2_O occurring at high-pT conditions is key to any quantitative evolutionary model of water-rich celestial bodies. In spite of the steady progress in diamond-anvil-cell and shock-wave technologies, the experimental investigation of transport properties of materials at planetary conditions is still challenging. In the specific case of H_2_O, the electrical conductivity is only known with large uncertainties along the Hugoniot curve on a limited portion of the pT diagram, and nothing is known about the heat conductivity^6,12–14^.

Computer simulations may be our only handle on the properties of matter at physical conditions that cannot be achieved in the laboratory. In the case of water, they have allowed us to discover new phases^5^ and to predict their properties at extreme pT conditions^6,15^ over an ever broader portion of its phase diagram^16^. The diverse local chemical environments that characterize the different relevant phases of water make classical force fields unfit for an accurate simulation of their properties, and call for a full quantum-mechanical, ab initio (AI), treatment of the chemical bond. Some transport properties of water at high-pT conditions, such as ionic (H and O) diffusivities and the electrical conductivity have indeed been estimated using AI molecular dynamics (AIMD) simulations^17^ and the Green-Kubo (GK) theory of linear response^18–21^. However, it has long and widely been argued that quantum-mechanical simulation methods could not be combined with the GK theory, because the latter is based on a microscopic representation of the energy (current) density, which is evidently ill-defined at a quantum-mechanical level^22^. The soundness of this objection, which would actually apply to a classical representation of the interatomic forces as well, was recently refuted for good by the introduction of a gauge invariance principle for transport coefficients^23–25^. In a nutshell, gauge invariance means that transport coefficients do not depend on the details of the microscopic representation of the conserved quantity being transported, as long as this representation sums to the correct value in the thermodynamic limit and its space correlations are short ranged. This remarkable finding implies that any (good, in the above sense) local representation of the energy leads to the same heat conductivity, thus paving the way to a fully ab initio treatment of heat transport^23^, which was recently generalized to multi-component systems^26^.

In this work we leverage these recent theoretical advances to estimate the thermal conductivity and other transport coefficients of stoichiometric H_2_O in the pT conditions to be found on ice-giant planets, like Uranus and Neptune, from equilibrium AIMD simulations, exploring its solid, PDL, and SI phases.

## Results

### Theory

Transport in macroscopic media is governed by the dynamics of hydrodynamic variables, i.e., by the long-wavelength components of the (current) densities of conserved extensive quantities^25,27,28^. For short, we will dub such densities conserved densities, the corresponding currents conserved currents, while the macroscopic averages of the latter will be called conserved fluxes. The GK theory of linear response^18–21^ states that transport coefficients (i.e., conductivities) are integrals of the various flux time autocorrelation functions, which, according to the Wiener–Khintchine theorem^29,30^, are the zero-frequency values of the corresponding power spectra. An important concept in the theory of transport is that of diffusive flux: we say that a flux is diffusive if its power spectrum does not vanish^25,26^ at zero frequency. Gauge invariance states that two different representations (“gauges”) of a same conserved density that differ by the divergence of a bounded vector field are equivalent in that they give rise to macroscopic fluxes whose difference is non-diffusive, thus resulting in the same conductivity^23,24^.

When addressing heat transport, the relevant conserved quantities are the energy and the numbers of particles (or masses) of each atomic species. Since the total-mass flux itself (i.e., the total momentum) is a constant of motion, for a *P*-species system the number of independent conserved fluxes is equal to *P* (energy, plus *P* − 1 partial masses). Further constraints may reduce the number of relevant conserved fluxes. For instance, in solids, such as ordinary ice, atoms do not diffuse and there cannot be any macroscopic mass flow: energy flux is therefore the only relevant one. In molecular liquids, such as ordinary water, the partial mass fluxes of each atomic species are non-diffusive if the molecules do not dissociate. This is so because the integral of the difference between the individual total momenta of different atomic species is bound by the finite variation of the molecular bond lengths^23,25^: also in this case, therefore, energy is the only relevant conserved quantity. On the contrary, the PDL and SI phases of water are truly multi-component systems, because the momentum of at least one atomic component is neither conserved nor is its integral bound by any molecular constraints.

Heat transport in multi-component systems has long been the subject of theoretical misconceptions and/or considered to be numerically intractable. For instance, the thermal conductivity is sometimes computed as the GK integral of the energy flux, **J**_*E*_: $\kappa \propto \mathop{\int}\nolimits_{0}^{\infty }\langle {{\bf{J}}}_{E}(t){{\bf{J}}}_{E}(0)\rangle dt$. This simplistic approach is manifestly wrong, as the resulting conductivity would depend on the arbitrary choice of the atomic formation energies. To see why this is so, let us consider the classical expression of the energy flux^25,31^: ${{\bf{J}}}_{E}=\frac{1}{\Omega }\left[\mathop{\sum }\nolimits_{n = 1}^{N}{{\bf{V}}}_{n}{\epsilon }_{n}+{\sum }_{n,m}({{\bf{R}}}_{n}-{{\bf{R}}}_{m}){{\bf{F}}}_{nm}\cdot {{\bf{V}}}_{n}\right],$where Ω is the system’s volume, **R**_*n*_, **V**_*n*_, and *ϵ*_*n*_ are the atomic positions, velocities, and energies, respectively, and **F**_*nm*_ = −∂*ϵ*_*m*_/∂**R**_*n*_ are interatomic forces. The heat conductivity cannot evidently depend on the arbitrary zero of the atomic energies. For instance, in ab initio calculations these energies differ in a pseudo-potential or in an all-electron scheme, whereas transport coefficients should not. A better choice would be to compute the heat conductivity from the GK integral of the heat flux, defined as ${{\bf{J}}}_{q}={{\bf{J}}}_{E}-\frac{1}{\Omega }\mathop{\sum }\nolimits_{S = 1}^{P}{h}_{S}{N}_{S}{\overline{{\bf{V}}}}_{S}$, where ${\overline{{\bf{V}}}}_{S}$ is the center-of-mass velocity and *h*_*S*_ the partial enthalpy of the *S*-th atomic species^32^. This approach has the advantage that **J**_*q*_ is no longer sensitive to a rigid shift in the atomic energies; it is still an approximation, though, as it neglects the coupling between energy and mass flow (Soret effect) in the calculation of *κ*. Even if, for several systems, it has been argued that the error in doing so is small^32^, this argument cannot be taken for granted a priori for any generic system. Furthermore, the calculation of partial enthalpies is rather involved^33–35^, and often the subject itself of crude approximations.

A rigorous methodology to deal with multi-component systems is provided by a combination of Onsager’s phenomenological approach^36,37^ and the GK theory of linear response^18–21^. In this approach the interactions among different conserved fluxes are explicitly accounted for by Onsager’s phenomenological relations:

1 $${J}_{i}\;=\;\sum _{j}{\Lambda }_{ij}\,{f}_{j},$$

where *J* is a generic conserved flux, *f* a thermodynamic affinity, i.e., the average gradient of the intensive variable conjugate to a conserved quantity, Λ is the matrix of Onsager’s phenomenological coefficients, and the suffixes enumerate in principle both different conserved quantities and the Cartesian components of their fluxes/affinities. In practice, in the following we will dispose of Cartesian components, and only enumerate different conserved fluxes/affinities, given that we will only be concerned with isotropic or cubic systems. Within the GK theory, and the Λ coefficients are expressed as integrals of the time correlation functions of the relevant fluxes:

2 $${\Lambda }_{ij}=\frac{\Omega }{{k}_{B}}\mathop{\int}\nolimits_{0}^{\infty }\left\langle {{\mathcal{J}}}_{i}(t){{\mathcal{J}}}_{j}(0)\right\rangle dt,$$

where ${{\mathcal{J}}}_{i}(t)$ is the time series of the *i*-th flux, *k*_*B*_ is the Boltzmann constant, and 〈 ⋅ 〉 indicates an equilibrium average. From now on, calligraphic fonts indicate samples of stochastic processes. The thermal conductivity is defined as the ratio between the energy flux and the temperature gradient, when all the other conserved fluxes vanish. In a two-component system this condition leads to the following expression for the heat conductivity:

3 $$\kappa =\frac{1}{{T}^{2}}\left[{\Lambda }_{EE}-\frac{{\left|{\Lambda }_{EM}\right|}^{2}}{{\Lambda }_{MM}}\right],$$

where the *M* suffix indicates the mass flux of one of the two components. The expression in square brackets is the inverse of the *EE* matrix element of the inverse of the 2 × 2 matrix of the Onsager coefficients. In the general, multivariate, case, the heat conductivity is proportional to the Schur complement of the mass block in Λ. In ref. ^26^ we have shown that this expression for the heat conductivity is invariant under the addition of an arbitrary linear combination of conserved fluxes (such as mass or adiabatic electronic charge) to the energy flux, and we named this further remarkable property of transport coefficients convective invariance.

Equation (3) shows that this procedure is numerically ill-conditioned, because the estimator of the integral in Eq. (2) becomes a random walk as a function of the upper limit of integration, as soon as the integrand has exhausted all its weight, thus making the expression in Eq. (3) singular whenever the estimator of the denominator vanishes^38–41^. A solution to this problem is provided by multivariate cepstral analysis^26^, briefly sketched below. According to the Wiener–Khintchine theorem^29,30^, the Onsager coefficients in Eq. (2) are proportional to the zero-frequency values of the flux cross power spectral density, ${S}_{ij}(\omega )=\mathop{\int}\nolimits_{-\infty }^{\infty }\langle {{\mathcal{J}}}_{i}(t){{\mathcal{J}}}_{j}(0)\rangle {{\rm{e}}}^{i\omega t}dt$:

4 $${\Lambda }_{ij}=\frac{\Omega }{2{k}_{B}}{S}_{ij}(\omega =0)$$

5 $${S}_{ij}(\omega )={\mathop{\mathrm{lim}}\limits_{\tau \to \infty }}\langle {{\mathcal{S}}}_{ij}^{\tau }(\omega )\rangle$$

6 $${{\mathcal{S}}}_{ij}^{\tau }(\omega )=\frac{1}{\tau }{\tilde{{\mathcal{J}}}}_{i}^{\tau }{(\omega )}^{* }\cdot {\tilde{{\mathcal{J}}}}_{j}^{\tau }(\omega )$$

7 $${\tilde{{\mathcal{J}}}}_{j}^{\tau }(\omega )=\mathop{\int}\nolimits_{0}^{\tau }{{\mathcal{J}}}_{j}(t){{\rm{e}}}^{i\omega t}dt.$$

The continuity and smoothness of the power spectrum at low frequency can be leveraged to systematically reduce the noise affecting the estimator of its zero-frequency value, as explained below. According to the central-limit theorem, the flux processes, ${{\mathcal{J}}}_{i}(t)$, are Gaussian because they are the space integrals of current densities, whose correlations are short ranged. Stationarity implies that their Fourier transforms, Eq. (7), are normal deviates that for large *τ* are uncorrelated for $\omega \;\ne \;\omega ^{\prime}$. It follows that the sample spectrum of Eq. (6), aka the cross-periodogram, is a collection of complex Wishart random matrices^42^ that are uncorrelated among themselves for different frequencies. Now, the Schur complement of a block of dimension *P* − 1 in a Wishart matrix of order *P* is proportional to a *χ*^2^ stochastic variable^26,42^. We conclude that the Schur complement of the mass block, ${{\mathcal{S}}}_{E}^{\prime}$, in the cross-periodogram given by Eq. (6), is the product of a smooth function of frequency, whose *ω* → 0 limit is the thermal conductivity we are after, times a set of independent, identically distributed, *χ*^2^ stochastic variables. By applying a low-pass filter to the logarithm of this quantity, one obtains a consistent estimator of the logarithm of the conductivity, as explained in ref. ^26^, a procedure that is known as cepstral analysis in sound engineering and speech recognition applications^43^.

### Simulations

**Fig. 1:**
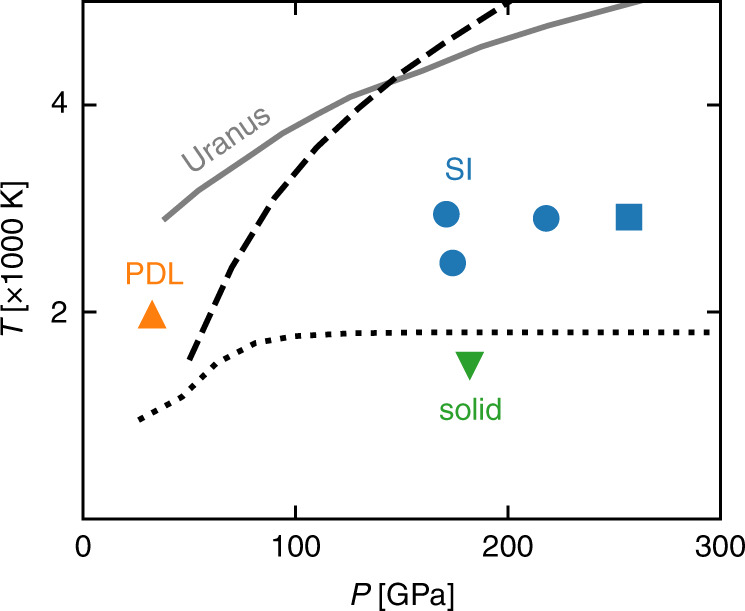
High-pT phase diagram of water. Shown are the ice-SI (dotted) and SI-PDL curves (dashed), and Uranus’ isentrope (gray solid). The symbols indicate the pT conditions at which the simulations were run.

The heat and charge transport properties of different (solid, PDL, and SI) phases of water in the 1000–3000 K and 30–250 GPa pT range have been explored by Car-Parrinello (CP) ab initio NVE molecular dynamics^44^, using the QUANTUM ESPRESSO suite of computer codes^45,46^. We believe that the CP Lagrangian formalism is particularly fit for transport simulations because the accurate conservation of the (extended) total energy allows one to generate long and stable trajectories without using thermostats. Figure 1 shows the phase diagram of water in such pT range. The SI-PDL (dashed) and ice-SI (dotted) phase boundaries are obtained from state-of-the-art shock-compression experiments^6^; Uranus’ isentrope (solid gray) from ab initio simulations^47^ is also reported. We have verified that a body-centered-cubic (BCC) to face-centered-cubic (FCC) transition in the oxygen lattice occurs for the SI phase at *P* ≈ 240 GPa and *T* ≈  3000 K, in accordance with recent theoretical^16^ and experimental findings^48^. We then ran three simulations for the BCC-SI phase (blue circles) and one for the FCC-SI one (blue square). We also ran a simulation for solid ice X (green triangle) and a simulation for the PDL (orange triangle) at pT conditions where the fraction of dissociated molecules is  ~10%^49^. We have explicitly checked that the electron energy gap computed along the various MD trajectories is always way larger than *k*_*B*_*T*, thus ruling out any direct electronic contributions to heat and charge transport. All the technical details of the simulations are reported in the Supplementary Note 1. Our results are summarized in Table 1.

**Table 1:** Summary of our results.

*T*, *P*, and *ρ* indicate temperature, pressure, and density, respetively. *κ*, *σ*, and *D* are thermal and electrical conductivities and atomic diffusivities, respectively. *σ*_*NE*_ is the value of the electric conductivity obtained from the Nernst–Einstein relation, Eq. (9).

## Discussion

**Fig. 2:**
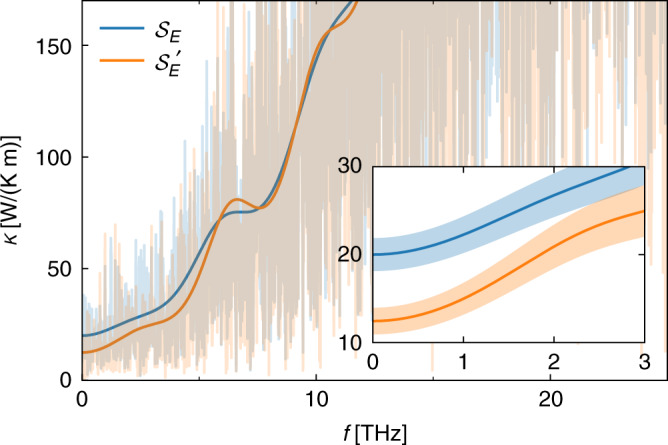
Energy-flux power spectra for FCC-SI water at average *T* = 2920 K and *P* = 257 GPa. Blue: plain periodogram, ${{\mathcal{S}}}_{E}$. Orange: reduced periodogram, ${{\mathcal{S}}}_{E}^{\prime}$, computed at vanishing mass fluxes, Eq. (3). The thick lines are the filtered spectra obtained via cepstral analysis. Inset: low-frequency zoom of with their estimated uncertainties.

We start the discussion of our results by highlighting the importance of a multi-component analysis of the heat- and mass-flux time series resulting from our simulations. In Fig. 2 we display the power spectrum of the energy flux of FCC-SI water at an average temperature *T* = 2920 ± 90 K and pressure *P* = 257 ± 2 GPa, evaluated according to two different prescriptions: blue lines refer to the plain spectrum of the energy flux computed within density-functional theory using the formulation of ref. ^23^; orange lines indicate the “residual spectrum” computed by assuming that the mass flux vanishes, according to Eq. (3). The sample power spectra (the “periodograms”) are displayed with faint lines, whereas those subject to cepstral filtering are displayed with thick lines; the latter are zoomed-in at low frequency and displayed in the inset, together with their statistical uncertainties. By looking at the zero-frequency value of the spectrum, cepstral analysis gives *κ* = 20 ± 2 W/(Km), and *κ* = 13 ± 2 W/(Km) neglecting and accounting for the interaction with the H mass flux, respectively. In effectively one-component systems, statistical analysis can be greatly facilitated by fixing a suitably defined optimal gauge for the diffusing current^50^. Since SI is a truly bicomponent system, a bivariate analysis is indeed needed to account for the interaction between different conserved fluxes and for a correct estimate of *κ*: considering the time series of the energy flux alone—as if the system were one-component—would overestimate the heat conductivity by 80%.

Convective invariance can also be leveraged to reduce the statistical noise, and thus the uncertainty, on the estimated value of *κ*, as explained in ref. ^26^. The addition of one or more components to the set of conserved fluxes to be analyzed decreases the total power of the reduced spectrum without affecting its value at zero frequency, thus making it smoother and the low-pass cepstral filter more efficacious. By adopting the adiabatic electron current as an additional flux, one obtains the refined result: *κ* = 12.8 ± 1.0 W/(Km). Further details on the statistical analysis of our data can be found in the Supplementary Information.

**Fig. 3:**
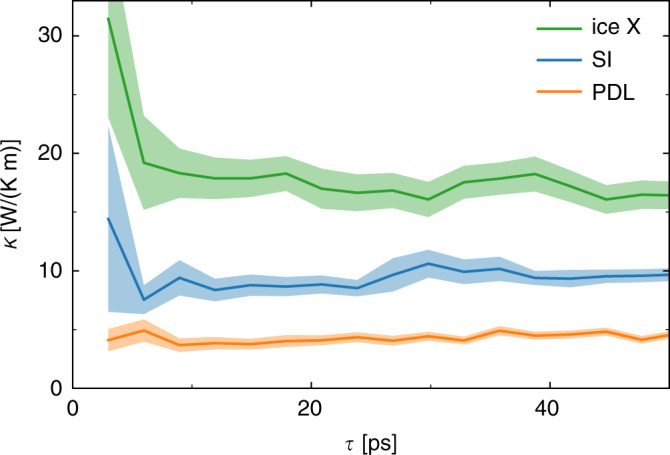
Thermal conductivity as a function of the length of the AIMD trajectory. Solid ice X (green), BCC-SI (*T* = 2470 K, *P* = 174 GPa, blue), and PDL (orange) phases of water, see Table 1. The shaded areas indicate the estimated statistical uncertainty.

Multi-component cepstral analysis, which has been performed using the thermocepstrum code^51^, allows us to obtain accurate transport coefficients from relatively short AIMD trajectories, particularly for the strongly anharmonic exotic phases of water occurring at the high-pT conditions of interest here. Figure 3 shows the values and the statistical uncertainties of the heat conductivity of different phases of water as a function of the length of the (reduced) energy-flux time series from which they are estimated. These data show that well-converged results with an uncertainty of  ≈15% are obtained with trajectories as short as 10–20 ps. Not surprisingly, the more crystalline a phase is, the larger the uncertainty for a same trajectory length (ice X > SI > PDL), due to the larger residual harmonicity of the structure. We stress that cepstral analysis is a self-averaging technique, in that the statistical error affecting the estimated conductivities can be accurately estimated and systematically reduced by increasing the length of the simulation, thus avoiding the need to average over different MD trajectories. Nonetheless, isotropy allows one to consider the three Cartesian components of the fluxes as different samples of a same process: the spectra have been thus averaged over Cartesian components.

Our results are summarized in Table 1. In the pT conditions examined here, the thermal conductivity of solid ice X is larger than that of the SI phase, which is itself larger than in PDL water. This is expected, again due to the decreasing level of harmonicity in going from a crystalline to a partially liquid and eventually fully liquid phase. We did not observe a significant dependence of *κ* upon the temperature for the SI phase in the explored range. The FCC-SI water has slightly larger heat conductivity than BCC-SI.

Pioneering AIMD simulations of charge transport in PDL water^17^ revealed that, rather unexpectedly, a classical model of charge conduction where hydrogen and oxygen ions carry an integer charge whose magnitudes equal their formal oxidation numbers (*q*_H_ = +1 and *q*_O_ = −2) yields the same conductivity that would be obtained from the exact quantum-mechanical expression of the electric current, based on Born’s effective charges. This surprising finding was given a solid theoretical foundation in a recent paper of ours where it was shown to result from the combined effects of gauge invariance of transport coefficients and topological quantization of adiabatic charge transport^52^. Leveraging this result, we computed the electrical conductivity from the cepstral analysis of the classical charge flux, defined as:

8 $${{\mathcal{J}}}_{Z}=\frac{1}{\Omega }\left({q}_{{\rm{H}}}\sum _{n\in {\rm{H}}}{{\mathcal{V}}}_{n}+{q}_{{\rm{O}}}\sum _{n\in {\rm{O}}}{{\mathcal{V}}}_{n}\right),$$

where the ${\mathcal{V}}$’s are ionic velocities.

The electrical conductivities resulting from our simulations are reported in Table 1. The data tagged with the “*NE*” subscript are obtained using the Nernst–Einstein equation^53^, which neglects all interionic correlations and that in the one-component case reads:

9 $${\sigma }_{NE}=\frac{{e}^{2}{q}_{{\rm{H}}}^{2}{N}_{{\rm{H}}}{D}_{{\rm{H}}}}{\Omega {k}_{B}T},$$

where *N*_H_ and *D*_H_ are the number of hydrogen atoms and their diffusivity, respectively. In the case of PDL, Eq. (9) hardly applies, as it would depend on too large a number of parameters (the concentrations, life-times, and diffusivities of the various ionic charge carriers). Our results are consistent with previous theoretical estimates^16,17^, as well as with the experimental data obtained from electrical impedance measurements along the liquid or precompressed Hugoniot^12–14^, summarized in Fig. 4 of ref. ^6^: *σ* ~ 150  S/cm for the SI phase in the range 100–150 GPa and 2000–3000 K; and *σ* ~ 30 S/cm for the PDL phase at ≈30 GPa and 2000 K. Two important trends emerge from our results. First, the NE relation severely underestimates the conductivity in SI water, as already observed in other SI systems^53^. At variance with these findings, when charge carriers of opposite signs coexist in an electrolyte, the short-range correlations among them may screen the amount of transported charge, thus determining a decrease of the electric conductivity with respect to the predictions of the NE approximation^54^. In the second place, the electrical conductivity in the FCC-SI phase is sensitively larger than in the BCC one, in contrast to the opposite trend displayed by hydrogen diffusivity, which are instead slightly smaller in the FCC phase, thus resulting in comparable predictions for the two phases of the NE approximation (*σ*_*NE*_). The lesser ability of the NE approximation to predict the conductivity in the FCC than in the BCC phase indicates a stronger effect of interionic correlations in the former case: the higher energy barriers for a single proton hop in FCC—due to its larger packing density^55^, and resulting in a slightly smaller ionic diffusivity—may be effectively decreased by a cooperative motion of two or more protons (as already observed for the carrier dynamics in solid-state electrolytes^56^), and thus lead to an overall larger electrical conductivity.

In this paper we have reported on the first theoretically rigorous and numerically accurate evaluation of the thermal and electric conductivities of various phases of water occurring at the pressure and temperature conditions to be found in the interior of ice-giant planets, made possible by recent advances in transport theory and data analysis. In the case of the heat conductivity, our results set a reference in the wide range of values used in evolution models of Uranus and Neptune^57^ or given by recent MD-based estimates on dissociating water^58^, and their moderate values point towards more efficient trapping of heat in the deep interior of these planets. These results have been instrumental in the development of a novel model of the thermal evolution of Uranus, featuring a frozen core and an anomalously low heat flow, resulting in the observed low luminosity of this planet^59^. Finally, the electrical conductivity that we find for SI ice is far larger than assumed in previous models of the generation of the magnetic fields in Uranus and Neptune^60^. Since SI ice is likely to dominate the deeper sluggish layer that underlies the shallow fluid outer layer in which the magnetic field is produced, the large electrical conductivity of the SI phase can have a substantial impact on the geometry and time evolution of the magnetic field of these planets.

## Supplementary information

Supplementary Information

## Data Availability

The data that support the plots and relevant results within this paper are available on the Materials Cloud Platform at 10.24435/materialscloud:hn-6f.

## Code availability

Computer codes are available and freely downloadable from the QUANTUM ESPRESSO site and the Thermocepstrum GitHub page referenced below.
