# Supplementary material for: Heat and charge transport in H2O at ice-giant conditions from ab initio molecular dynamics simulations
Source: Nat Commun. 2020 Jul 17;11:3605. doi: 10.1038/s41467-020-17275-5 (PMC7367872; doi:10.1038/s41467-020-17275-5)
Supplement: Supplementary file 1 — Supplementary Information [file 41467_2020_17275_MOESM1_ESM.pdf]

Supplementary Information to "Heat and charge transport in H_2O at ice-giant conditions from ab initio molecular dynamics simulations"

Federico Grasselli,[1] Lars Stixrude,[2] and Stefano Baroni[1,3,*]

[1] *SISSA – Scuola Internazionale Superiore di Studi Avanzati, Trieste, Italy*
[2] *Department of Earth, Planetary, and Space Sciences, University of California Los Angeles, USA*
[3] *CNR – Istituto Officina dei Materiali, SISSA, 34136 Trieste*

[*] baroni@sissa.it

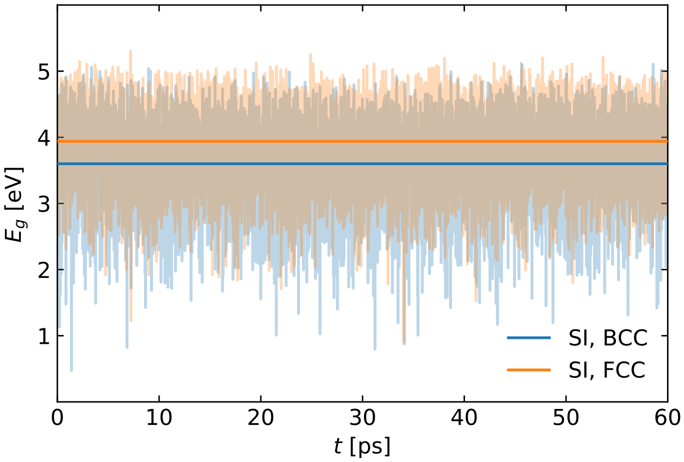

Supplementary Figure 1. Energy band gap, E_g, during the CP AIMD trajectory (transparent), and its average (solid horizontal lines). Values for the superionic phase with oxygen BCC lattice (blue, 2950 K, 171 GPa) and FCC lattice (orange, 2920 K, 257 GPa).

SUPPLEMENTARY NOTE 1. AB INITIO SIMULATIONS

Ab initio molecular dynamics simulations were run using the plane-wave pseudo-potential method and the Car-Parrinello (CP) Lagrangian formalism [1], as implemented in the `cp.x` component of the Quantum ESPRESSO suite of computer codes [2, 3]. We adopted norm-conserving pseudopotentials [4] from Ref. 5, which are accurate also at the present pT-conditions [6], and the PBE energy functional [7]. We employed a plane-wave energy cutoff of $E_{\mathrm{cut}} = 110\,\mathrm{Ry}$ for wave-functions and four times as much for the charge density. The fictitious electronic mass was set to 25 electronic masses and the integration time step to ≈ 0.0484 fs. These choices ensure adequate adiabatic decoupling of the electron fictitious dynamics from the ionic motion and conservation of the extended total energy in the relevant pT range considered here. All our simulations have been performed neglecting nuclear quantum effects (NQEs), as these are expected to become negligible as temperature increases. Even though NQEs may be counter-enhanced by high pressure [8], their account is beyond the scope of the present work.

Flux time series were collected from NVE simulations for at least 60 ps, after an initial NPT equilibration of a few ps at the target pT conditions, followed by $\sim 4\,\mathrm{ps}$ of further NVE equilibration. NPT simulations were run with a chain of three Nosé-Hoover thermostats [9] with a frequency of 60 THz, while pressure was controlled via a Parrinello-Rahman barostat [10]. In order to minimize the effects of Pulay stresses, the kinetic energy functional was modified as: $\mathbf{G}^2 \mapsto \mathbf{G}^2 + A\left[1 + \mathrm{erf}[(\mathbf{G}^2 - E_0)/\sigma]\right]$ [11] with $E_0 = 100\,\mathrm{Ry}$, $A = 170\,\mathrm{Ry}$, and $\sigma = 15\,\mathrm{Ry}$.

Our simulations were run on samples of 128 atoms for ice-X, SI-BCC, and PDL water, and of 108 atoms for SI-FCC. In order to estimate the magnitude of finite-size effects arising in the AIMD simulation of the thermal conductivity, we have performed extensive MD simulations of PDL water samples of 128, 256, 512, and 1024 H_2O units, using a deep-neural-network potential trained on our ab initio trajectory [12], at the same conditions ($T \approx 1970$ K and $p \approx 33$ GPa). These results indicate that finite-size corrections to the thermal conductivity computed for our sample of 128 H_2O units should be smaller than 5%, in qualitative agreement with systematic tests performed in Ref. 13.

SUPPLEMENTARY NOTE 2. FLUX TIME SERIES

The energy and charge fluxes, $\mathcal{J}_E(t)$ and $\mathcal{J}_Z(t)$, were sampled every 30 or 40 time steps of the original CP simulation ($\Delta_t = 1.45\,\mathrm{fs}$ or $1.94\,\mathrm{fs}$), depending on the specific simulation. This sampling rate is high enough to make the flux power spectra vanish at the corresponding Nyqvist frequency, $f_{\mathrm{Ny}} = 1/(2\Delta_t)$, thus avoiding any aliasing effects. We verified that the energy band gap is far larger than $k_B T$ all along our simulations, thus excluding any possible non adiabatic effects in heat and charge transport at the pT conditions of interest here. The time series of the energy gap occurring in two different SI phases of water are reported in Supplementary Figure 1.

The energy flux was estimated using the expression derived in Ref. 14. The charge flux was estimated using two different expressions: *i)* the charge flux, $\mathcal{J}_Z$, estimated from the classical expression derived from the formal oxidation numbers $q_{\mathrm{H}} = +1$ and $q_{\mathrm{O}} = -2$, according to the topological theory of adiabatic charge transport introduced in Ref. 15, and *ii)* the standard quantum-mechanical one, $\mathcal{J}'_Z$, that is the sum of the adiabatic electron-charge flux, $\mathcal{J}_{el}$, and

phase	N_{H_2O}	ρ [g/cm^3]	T [K]	p [GPa]	f^* [THz]	P^*	κ [W/(Km)]	σ [S/cm]	D [Å^2/ps]
ice X	128	3.52	1488 ± 45	182 ± 1	13.8	6	16.1 ± 1.1	–	consist. zero
SI^{BCC}	128	3.39	2474 ± 78	174 ± 2	31.3	3	9.4 ± 0.6	135 ± 7	4.88 ± 0.13 (H)
SI^{BCC}	128	3.35	2945 ± 88	171 ± 2	36.9	5	10.7 ± 0.7	180 ± 5	7.41 ± 0.12 (H)
SI^{BCC}	128	3.61	2905 ± 86	218 ± 2	31.3	3	9.9 ± 0.7	198 ± 9	7.38 ± 0.16 (H)
SI^{FCC}	108	3.82	2917 ± 93	257 ± 2	38.3	5	12.8 ± 1.0	256 ± 8	7.17 ± 0.13 (H)
PDL	128	2.04	1970 ± 60	33 ± 1	49.2	5	4.1 ± 0.3	42 ± 3	3.10 ± 0.03 (H)<br>0.92 ± 0.02 (O)
d-ice X	127	3.49	1523 ± 47	179 ± 1	18.1	8	17.0 ± 1.5	< 0.1	0.0039 ± 0.0006 (H)
d-SI^{BCC}	127	3.49	2302 ± 94	190 ± 2	38.3	6	11.6 ± 0.9	95 ± 3	3.04 ± 0.04 (H)<br>0.0085 ± 0.0006 (O)

Supplementary Table 1. Results of the different NVE simulations. N_{H_2O} is the number of H_2O stoichiometric units employed in the simulation. Temperature and pressures are reported together with their standard deviation. The last two simulations concern the defected structure, where one H_2O was removed. The temperatures and pressures are reported with their standard deviations. The uncertainty on the diffusivity is estimated via a 6 block analysis with $\approx$ 10-ps segments.

the classical current of the atomic cores ($Z_H = +1$ and $Z_O = +6$). The two expressions read:

$$\mathcal{J}_Z = \frac{e}{\Omega}\left(q_H \sum_{n\in H} \mathcal{V}_n + q_O \sum_{n\in O} \mathcal{V}_n \right) \tag{1}$$

$$\mathcal{J}'_Z = \frac{e}{\Omega}\left(Z_H \sum_{n\in H} \mathcal{V}_n + Z_O \sum_{n\in O} \mathcal{V}_n \right) + \mathcal{J}_{el}. \tag{2}$$

The adiabatic electron charge current, $\mathcal{J}_{el}$, can be computed as:

$$\mathcal{J}_{el} = \frac{-2e}{\Omega} \mathfrak{Re} \sum_v \langle \bar{\phi}^c_v | \dot{\phi}^c_v \rangle, \tag{3}$$

where the sum runs over the occupied Kohn-Sham states, $\{\phi_v\}$, and the orbitals

$$\begin{aligned} |\bar{\phi}^c_v\rangle &= \hat{P}_c \, \mathbf{r} \, |\phi_v\rangle, \\ |\dot{\phi}^c_v\rangle &= \dot{\hat{P}}_v \, |\phi_v\rangle, \end{aligned} \tag{4}$$

are the projections over the empty-state manifold of the action of the position operator over the v-th occupied orbital, and of its adiabatic time derivative [3]. The operators $\hat{P}_v$ and $\hat{P}_c = 1 - \hat{P}_v$ are projectors over the occupied- and empty-states manifolds, respectively. Equations (4) are well defined in periodic boundary conditions and have been computed from standard density-functional perturbation theory [16].

SUPPLEMENTARY NOTE 3. CEPSTRAL ANALYSIS

Transport coefficients were estimated by cepstral analysis of the flux time series, as explained in Refs. 17 and 18, using the `thermocepstrum` code [19]. This technique allows one to obtain accurate transport coefficients, as well as a quantitative estimate of their statistical accuracy, depending on two parameters: an effective Nyqvist frequency, f^*, used to limit the analysis to a properly defined low-frequency portion of the spectrum, and the number P^* of inverse Fourier ("*cepstral*") coefficients of the logarithm of the spectrum in the low-frequency region thus defined. The estimated conductivities depend very little on f^*, whereas an optimal value of P^* can be estimated in most cases by statistical model-selection techniques, as explained in Ref. 17. In our applications P^* is determined via the so-called *Akaike Information Criterion* [17, 20]

Supplementary Figure 2 displays the sample power spectra of $\mathcal{J}_{el}$, $\mathcal{J}_Z$ and $\mathcal{J}'_Z$, scaled in units such that the zero-frequency value is the electrical conductivity in S/cm. The faint lines are obtained by applying a moving average filter [21] with a window of 0.1 THz to the raw periodograms; the solid smooth lines are obtained by applying a cepstral filter [17] with the parameters reported in Supplementary Table 1. The definitions $\mathcal{J}_Z$ and $\mathcal{J}'_Z$, lead to the same

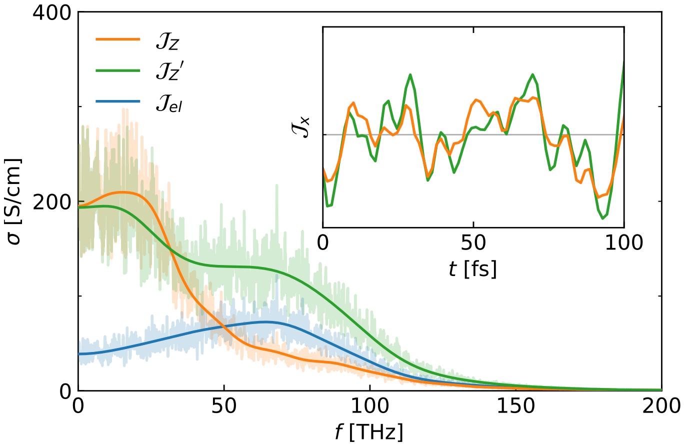

Supplementary Figure 2. Periodograms (filtered with a 0.1 THz window) and cepstral analysis of electric fluxes, as defined in Eqs. (1), (2) and (3) and following, for the simulation of the SI-BCC phase at $T = 2910\,\mathrm{K}$ and $P = 218\,\mathrm{GPa}$. Inset: a sample of the time series of the the electric fluxes ($x-$component), whose zero is highlighted by the horizontal gray line.

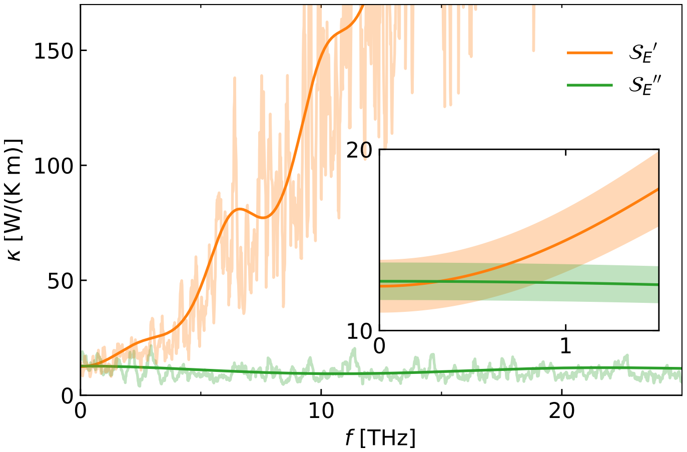

Supplementary Figure 3. Energy-flux power spectra for FCC super-ionic water at average $T = 2920\,\mathrm{K}$ and $P = 257\,\mathrm{GPa}$. Orange: 2-variate reduced periodogram, $\mathcal{S}'_E$. Green: 3-variate reduced periodogram obtained under the constraint of vanishing mass and adiabatic electronic fluxes, $\mathcal{S}''_E$. The reported data are the result of a 0.2 THz window filtering. The smooth thick lines are the filtered spectra obtained via cepstral analysis. Inset: low-frequency zoom of with their estimated uncertainties.

conductivity, even if their time series values differ, as is clear from the inset, in agreement with Ref. [15], where this finding was proved to be the consequence of gauge-invariance of transport coefficients and topological quantization of adiabatic charge transport in electronically gapped materials.

As stated in the main text, a 3-variate analysis on thermal conductivity adopting the adiabatic electron flux $\mathcal{J}_{el}$ as an additional flux reduces the statistical uncertainty without affecting the estimate of the thermal conductivity, as shown in Supplementary Figure 3 (green). The 3-variate analysis produces a flatter spectrum featuring a reduced total power, corresponding to a smaller number of cepstral coefficients, P^*, thus ensuring a smaller statistical error.

A comprehensive summary of our results, along with the values of f^* and P^* used for cepstral analysis, is given in Supplementary Table 1.

SUPPLEMENTARY NOTE 4. DEFECTED STRUCTURE

We also performed two further simulations—one in solid ice X and one in the SI phase with BCC oxygen structure—for defected structures where two H and one O atoms were removed, to preserve the stoichiometry of the ideal systems, resulting in a defect relative concentration of $1/128 \approx 0.008$. In the SI phase, vacancy hopping in the oxygen lattice is observed in the form of jumps of one or more than one O atoms within the same hopping event. From the mean

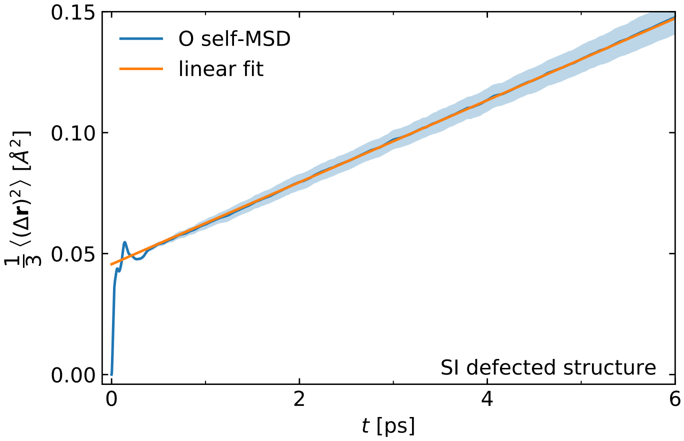

Supplementary Figure 4. Mean square displacement of oxygen atoms (blue solid) and its statistical uncertainty from a 6 block analysis of 10ps segments.

square displacement of oxygen atoms with respect to their center of mass, we estimated the O self-diffusion coefficient as $D_{\mathrm{O}} \approx (8.5 \pm 0.6) \times 10^{-3}$ Å^2/ps. The linear growth of the oxygen mean square displacement, together with the related statistical uncertainty from a 6-block analysis of 10ps segments, is reported in Supplementary Figure 4. On the contrary, in the defected ice X structure below the SI transition we observed no oxygen self-diffusion.

Even if such defect concentration is far larger than what it is expected to be at the pT-conditions of Uranus and Neptune from extrapolation of experimental data [22], it is still insufficient to strongly affect the thermal conductivity, when compared to the ideal-structure value. In fact, we obtained values for κ which are compatible with those of the ideal structures, namely $\kappa \approx 17$ and $\kappa \approx 12$ W/(Km) for the solid and the SI phases, respectively.

SUPPLEMENTARY REFERENCES

[1] R. Car and M. Parrinello, "Unified approach for molecular dynamics and density-functional theory," Phys. Rev. Lett. **55**, 2471–2474 (1985).

[2] P Giannozzi, S Baroni, N Bonini, M Calandra, R Car, C Cavazzoni, D Ceresoli, G L Chiarotti, M Cococcioni, I Dabo, A D Corso, Sd Gironcoli, S Fabris, G Fratesi, R Gebauer, U Gerstmann, C Gougoussis, A Kokalj, M Lazzeri, L Martin-Samos, N Marzari, F Mauri, R Mazzarello, S Paolini, A Pasquarello, L Paulatto, C Sbraccia, S Scandolo, G Sclauzero, A P Seitsonen, A Smogunov, P Umari, and R M Wentzcovitch, "QUANTUM ESPRESSO: a modular and open-source software project for quantum simulations of materials," J. Phys. Condens. Matter **21**, 395502 (19pp) (2009).

[3] P Giannozzi, O Andreussi, T Brumme, O Bunau, M Buongiorno Nardelli, M Calandra, R Car, C Cavazzoni, D Ceresoli, M Cococcioni, N Colonna, I Carnimeo, A Dal Corso, S de Gironcoli, P Delugas, R A DiStasio Jr, A Ferretti, A Floris, G Fratesi, G Fugallo, R Gebauer, U Gerstmann, F Giustino, T Gorni, J Jia, M Kawamura, H-Y Ko, A Kokalj, E Küçükbenli, M Lazzeri, M Marsili, N Marzari, F Mauri, N L Nguyen, H-V Nguyen, A Otero de-la Roza, L Paulatto, S Poncé, D Rocca, R Sabatini, B Santra, M Schlipf, A P Seitsonen, A Smogunov, I Timrov, T Thonhauser, P Umari, N Vast, X Wu, and S Baroni, "Advanced capabilities for materials modelling with q uantum espresso," Journal of Physics: Condensed Matter **29**, 465901 (2017).

[4] David Vanderbilt, "Optimally smooth norm-conserving pseudopotentials," Phys. Rev. B **32**, 8412–8415 (1985).

[5] M Schlipf and F Gygi, "Optimization algorithm for the generation of oncv pseudopotentials," Computer Physics Communications **196**, 36 – 44 (2015), with pseudopotentials downloaded from `http://www.quantum-simulation.org/potentials/sg15_oncv/upf/`.

[6] Jiming Sun, Bryan K Clark, Salvatore Torquato, and Roberto Car, "The phase diagram of high-pressure superionic ice," Nature communications **6**, 8156 (2015).

[7] John P. Perdew, Kieron Burke, and Matthias Ernzerhof, "Generalized gradient approximation made simple," Phys. Rev. Lett. **77**, 3865–3868 (1996).

[8] Jiming Sun, *High Pressure Superionic Ice Phase Diagram*, Ph.D. thesis, Princeton University (2019).

[9] Shuichi Nosé, "A unified formulation of the constant temperature molecular dynamics methods," The Journal of chemical physics **81**, 511–519 (1984); William G Hoover, "Canonical dynamics: Equilibrium phase-space distributions," Physical review A **31**, 1695 (1985); Glenn J Martyna, Michael L Klein, and Mark Tuckerman, "Nosé-Hoover chains: The canonical ensemble via continuous dynamics," J. Chem. Phys. **97**, 2635–2643 (1992).

[10] M_ Parrinello and A Rahman, "Crystal structure and pair potentials: A molecular-dynamics study," Physical Review Letters **45**, 1196 (1980); Michele Parrinello and Aneesur Rahman, "Polymorphic transitions in single crystals: A new molecular dynamics method," Journal of Applied physics **52**, 7182–7190 (1981).

[11] M. Bernasconi, G.L. Chiarotti, P. Focher, S. Scandolo, E. Tosatti, and M. Parrinello, "First-principle-constant pressure molecular dynamics," Journal of Physics and Chemistry of Solids **56**, 501–505 (1995).

[12] Linfeng Zhang, Jiequn Han, Han Wang, Roberto Car, and Weinan E, "Deep potential molecular dynamics: A scalable model with the accuracy of quantum mechanics," Phys. Rev. Lett. **120**, 143001 (2018).

[13] Marcello Puligheddu and Giulia Galli, "Atomistic simulations of the thermal conductivity of liquids," Phys. Rev. Materials **4**, 053801 (2020).

[14] Aris Marcolongo, Paolo Umari, and Stefano Baroni, "Microscopic theory and ab initio simulation of atomic heat transport," Nature Phys. **12**, 80–84 (2016).

[15] Federico Grasselli and Stefano Baroni, "Topological quantisation and gauge-invariance of charge transport in liquid insulators," Nature Physics **15**, 967–972 (2019).

[16] S Baroni, S de Gironcoli, A Dal Corso, and P Giannozzi, "Phonons and related crystal properties from density-functional perturbation theory," Rev. Mod. Phys. **73**, 515–562 (2001).

[17] Loris Ercole, Aris Marcolongo, and Stefano Baroni, "Accurate thermal conductivities from optimally short molecular dynamics simulations," Sci. Rep. **7**, 15835 (2017).

[18] Riccardo Bertossa, Federico Grasselli, Loris Ercole, and Stefano Baroni, "Theory and numerical simulation of heat transport in multicomponent systems," Phys. Rev. Lett. **122**, 255901 (2019).

[19] Loris Ercole and Riccardo Bertossa, "`ThermoCepstrum`: a code to estimate transport coefficients from the cepstral analysis of a multi-variate current stationary time series," `https://github.com/lorisercole/thermocepstrum` (2017–2018).

[20] H. Akaike, *Information theory and an extension of the maximum likelihood principle, in 2nd International Symposium on Information Theory* (edited by B. N. Petrov and F. Csáki, 1972) pp. 267–281; "A new look at the statistical model identification," IEEE Trans. Autom. Control **19**, 716–723 (1974).

[21] Eric W. Weisstein, "Moving average," From MathWorld – a Wolfram Web Resource `http://mathworld.wolfram.com/MovingAverage.html`.

[22] DL Goldsby and David L Kohlstedt, "Superplastic deformation of ice: Experimental observations," Journal of Geophysical Research: Solid Earth **106**, 11017–11030 (2001).
